# Spatial Orientation and Morphology of the Pulmonary Artery: Relevance to Optimising Design and Positioning of a Continuous Pressure Monitoring Device

**DOI:** 10.1007/s12265-016-9690-4

**Published:** 2016-04-13

**Authors:** Su-Lin Lee, Heba Aguib, Julien Chapron, Reza Bahmanyar, Alessandro Borghi, Olive Murphy, Chris McLeod, Ahmed ElGuindy, Magdi Yacoub

**Affiliations:** The Hamlyn Centre, Imperial College London, Room 414A, Bessemer Building, South Kensington Campus, London, SW7 2AZ UK; Aswan Heart Centre, Magdi Yacoub Foundation, Aswan, Egypt; Qatar Cardiovascular Research Center, Doha, Qatar; Institute of Biomedical Engineering, Imperial College London, London, UK; Institute of Child Health, University College, London, UK; Analog Devices, Cork, Ireland; Harefield Heart Science Centre, National Heart & Lung Institute, Imperial College London, London, UK

**Keywords:** Pulmonary artery, Device design, Pressure monitoring, Orientation, Morphology

## Abstract

Personalised treatment of heart disease requires an understanding of the patient-specific characteristics, which can vary over time. A newly developed implantable surface acoustic wave pressure sensor, capable of continuous monitoring of the left ventricle filling pressure, is a novel device for personalised management of patients with heart disease. However, a one-size-fits-all approach to device sizing will affect its positioning within the pulmonary artery and its relationship to the interrogating device on the chest wall on a patient-specific level. In this paper, we analyse the spatial orientation and morphology of the pulmonary artery and its main branches in patients who could benefit from the device and normal controls. The results could optimise the design of the sensor, its stent, and importantly its placement, ensuring long-term monitoring in patient groups.

## Introduction

Heart failure is one of the leading causes of morbidity and mortality worldwide [1–4]. The disease is generally progressive, with the advanced forms of the disease carrying a very poor prognosis, and importantly, it is becoming more resistant to conventional therapy [5, 6]. Therapy is based on a combination of pharmaceutical agents, any of which can have side-effects which should be avoided. Therapy must therefore be personalised, taking into account patient-specific haemodynamics which can also vary over time. A principal control variable would ideally be pulmonary blood pressure as it usually reflects left ventricular filling pressures. Monitoring progression is therefore essential for improving outcome. Changes in filling pressures of the left ventricle (LV) have been shown to precede symptoms, making accurate measurements of these pressures both at rest and exercise a priority in managing these patients [7, 8].

Several implantable devices have been developed to monitor LV filling pressures noninvasively, either indirectly or directly [9–13]. The EndoSure device (CardioMems, St. Jude Medical, St Paul, MN, USA) [9, 10] can only be interrogated at intervals, as the recording system requires the patient to lie down, providing only a snapshot of information at rest. This is a major limitation as some patients show normal resting haemodynamics but with pathological elevation in LV filling pressures during exercise [14, 15]. This is most pertinent for patients with heart failure with a preserved ejection fraction (HFpEF) as well as patients with primary pulmonary hypertension (PH).

The only device which can provide continuous monitoring of the filling pressure at rest and during exercise is the implantable surface acoustic wave pressure sensor (SAWPS) [16] currently is being developed at Imperial College London. The sensor is designed with an antenna that is mounted on a nitinol stent to be placed in the patient’s pulmonary artery (PA). The accuracy and reliability of the measurement critically depends on the position of the sensor in the pulmonary artery and its relationship to the interrogating device (ID) on the chest wall. Accurate characterization of the various morphologies of the PA and its branches, as well as its relation to the chest wall, therefore, is critical to the development and placement of the monitoring device.

The goal of this study is to analyse spatial orientation and morphology across different patient populations. While studies on the measurements of the PA diameter at the level of the bifurcation have been performed [17], the examination of how the diameter and orientation changes across the PA length has not been considered. Additionally, choosing the ideal location of the ID on the chest wall to improve signal and reading is addressed in detail. We studied the PAs of 25 individuals (20 candidates with heart failure or pulmonary arterial hypertension and 5 normal controls) and performed initial experiments in virtual stent fitting and preclinical deployment.

## Methods

### SAWPS Device

**Fig. 1:**
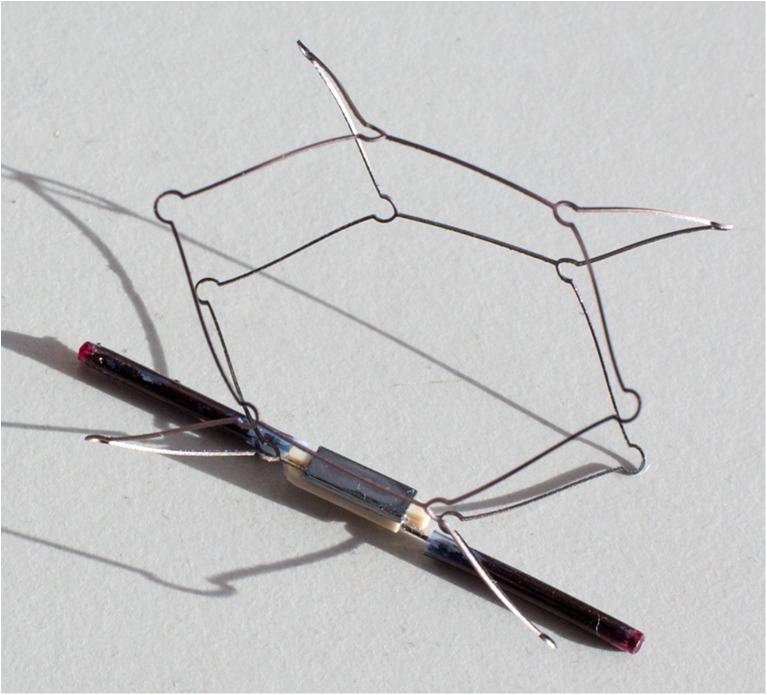
The surface acoustic wave sensor (SAWPS)

The surface acoustic wave pressure sensor (SAWPS) is a novel wireless transponder used in conjunction with a miniature communication system for continuous evaluation of PA pressure in patients with severe heart failure and those with pulmonary hypertension. It allows for accurate measurements to be taken on a continuous basis, and then transmitted remotely, allowing for blood pressure to be continuously monitored throughout waking and sleeping activities [18]. The implantable SAWPS components are an antenna and a passive, battery-less sensor fixed on an expandable Nitinol stent as shown in Fig. 1. When implanted, accurate PA pressure measurements from the sensor are transferred to an external interrogation system, which on the one hand powers the sensor and on the other hand receives, interprets, retransmits, and stores the acquired signal.

Previous studies by Murphy et al. [16, 18] were conducted to specify design, material properties and mechanical characteristics of the sensor, antenna and interrogator/transmitter to assure sufficient wave propagation and signal transfer. The antenna is designed to have a suitable shape and the required radiation efficiency for use in the PA and the length of the antenna from tip to tip is currently set to 37 mm. Additionally, finite element modelling (FEM) and experiments were conducted to identify arterial stress patterns and stent radial forces for different stent strut thickness [4].

### Study Population

The study includes normal subjects and a representative sample of the patients who could potentially benefit from the SAWPS device (Table 1). 15 heart failure (HF) patients, grouped according to the New York Heart Association (NYHA) classes [19] (5 NYHA Class II, 5 NYHA Class III and 5 NYHA Class IV), who were admitted in the Aswan Heart Centre (AHC) Heart Failure Clinic, and 5 patients with pulmonary hypertension (groups 1 and 4) underwent clinical investigation. 5 healthy subjects were included in the normal cohort. The average age and body surface area (BSA) for each group were: 58 ± 11/1.83 ± 0.21 m^2^ for NYHA Class II patients, 51 ± 8/1.86 ± 0.20 m^2^ for NYHA Class III, 62 ± 10/2.07 ± 0.25 m^2^ for NYHA Class IV and 30 ± 5/1.84 ± 0.37 m^2^ for PH and 32 ± 3/1.92 ± 0.05 m^2^ for normal subjects.

**Table 1:** Clinical data of the subjects included in this study

Group	Gender	Age	Weight (kg)	Height (cm)	BSA (m^2^)
HF NYHA Class II	F	69	63	154	1.64
	M	61	88	173	2.05
	M	65	60	168	1.67
	M	55	85	180	2.06
	F	42	65	171	1.75
HF NYHA Class III	M	43	89	168	2.04
	F	62	61	158	1.64
	F	53	75	170	1.88
	F	55	64	157	1.67
	M	43	93	168	2.08
HF NYHA Class IV	F	61	90	160	2.00
	F	78	66	152	166
	M	63	97	180	2.20
	M	58	110	170	2.28
	M	50	105	170	2.23
PH	M	26	131	170	2.49
	F	28	74	155	1.78
	F	28	65	156	1.68
	F	39	69	156	1.73
	F	29	54	157	1.53
Normal	M	33	79	177	1.97
	M	27	80	172	1.96
	M	43	75	176	1.91
	F	35	73	170	1.86
	F	29	77	166	1.88

*HF* heart failure, *NYHA* New York Heart Association functional classification, *PH* pulmonary hypertension

For each patient, images of the PA and chest wall structure were acquired. Selected heart failure and pulmonary hypertension patients had archived computed tomography (CT) images, scanned using a dual-source 128-slice FLASH Siemens CT scanner. The acquisition for heart failure patients followed a breath-hold, cardiac-gated scanning protocol with a slice thickness of 0.6 mm. Pulmonary hypertension patients had breath-hold, non-gated CT scans with a slice thickness of 1 mm. For the controls, gated scans were used.

All subjects’ data utilised in this study were based on data previously acquired for clinical purposes after approval from the Ethics Committee.

### 3D Analysis—Spatial Orientation and Morphology

**Fig. 2:**
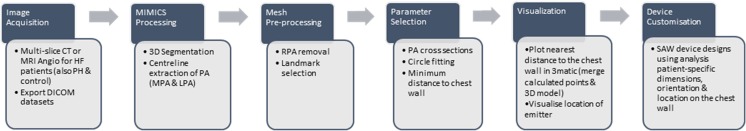
Processing flowchart for medical imaging derived 3D analysis of PA

To determine the range of PA sizes and orientations the SAWPS device must accommodate, 3D analysis of the PA was performed to extract the necessary parameters to ensure that the final device(s) will fit in the majority of patients. The workflow is shown in Fig. 2.

The DICOM images were imported into Mimics (Materialise, Leuven, Belgium) for segmentation of the PA (including left and right pulmonary arteries) and the chest wall. The models of the structures were then exported as triangular meshes in STL format. In addition, the centrelines of the PA models were automatically produced by Mimics and exported. The STL models were imported into MeshLab [20] to remove noise in the meshes through the use of Poisson mesh regeneration; these final meshes were exported for final measurements and analysis in MATLAB.

The proximal section of the PA was divided into two main segments. The first was the main pulmonary artery (MPA) leading to the left pulmonary artery (LPA), as shown in Table 2. The second was the right pulmonary artery (RPA), treated as a branch off the first structure. Along the length of the MPA-LPA centrelines, four main landmarks were manually identified: the pulmonary valve, the beginning of the RPA branch off the MPA, the end of the RPA branch off the MPA, and the first branch of the LPA. The analysis of the MPA-LPA was from the pulmonary valve to the first branch of the LPA. The analysis of the RPA was from the branch off the MPA and terminated at its first branch.

**Table 2:**
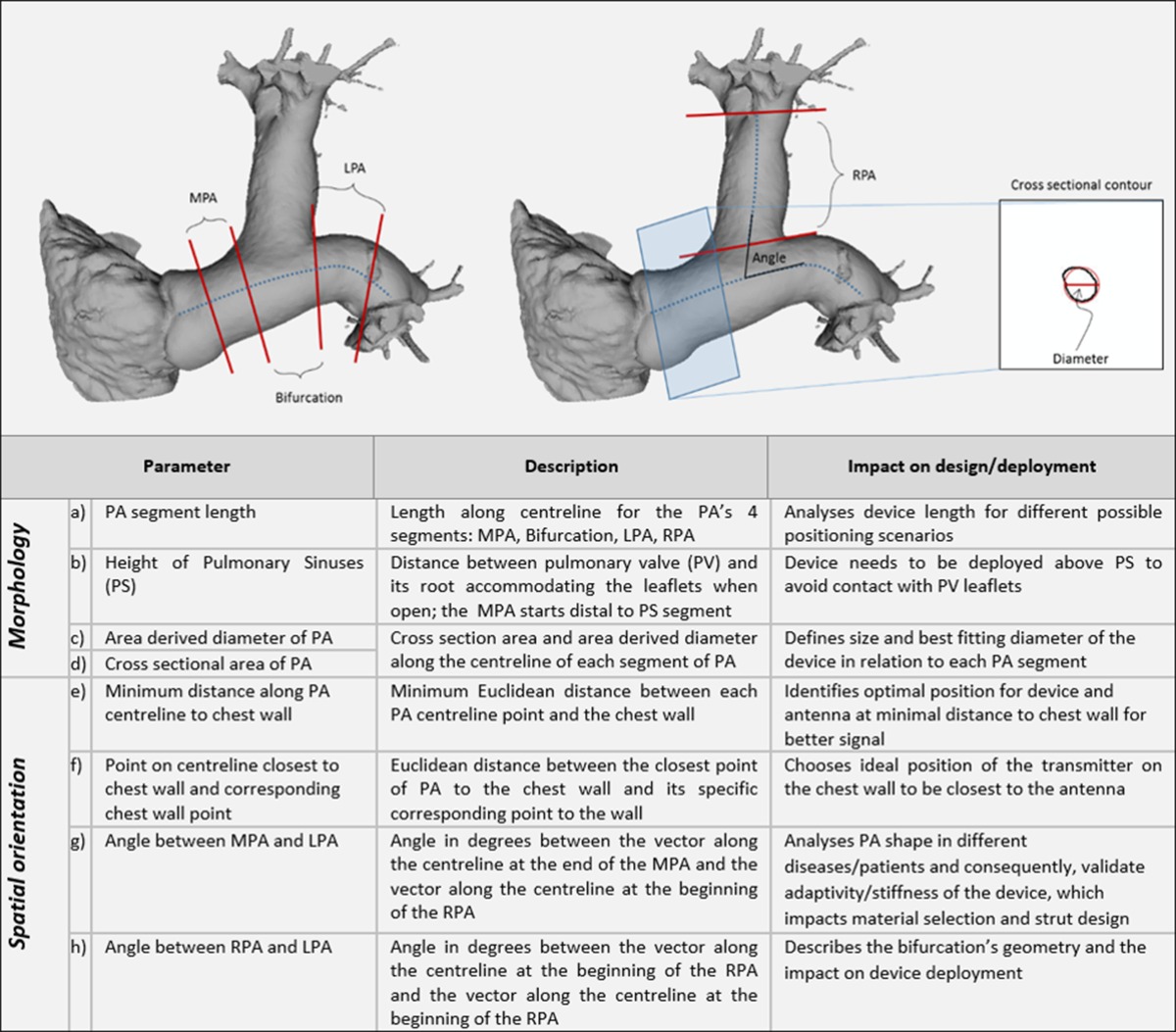
Parameters and segments of the PA for personalised SAW device design optimization

To attempt to normalise PA centreline lengths, the centreline for each MPA-LPA structure was linearly interpolated and the MPA and LPA were sampled to 50 points each, both separated by the bifurcation that leads to the RPA. Likewise, the centreline for each RPA structure was linearly interpolated and sampled to 40 points, with the number of points chosen empirically. These points were ordered and the lengths along the PA segments (as described in Table 2) were calculated as the lengths of their corresponding centrelines. The vector was defined by the line segments $\overset{\rightharpoonup }{P_i{P}_{i+1}}$, where *P*_*i*_ is the *i*th point in the centreline, was used as the normal to the *i*th orthogonal cutting plane. There was one orthogonal cutting plane for each point in the centrelines.

For each centreline point, the intersections of the cutting plane with the surface mesh of the PA were calculated and the resulting line segments were ordered into a contour. The area of each contour corresponded to the cross sectional area of the PA. A circle was also fitted to the points in this contour [21] and the diameter of this circle was used as the approximation of the diameter of the PA. For each centreline point, the minimum Euclidean distance from the point to the chest wall was recorded; likewise, the closest point on the chest wall was recorded.

Finally, to examine the orientation of the different parts of the PA with respect to each other, vectors were defined along the lengths of the beginning of the RPA branch, the end of the MPA, and the beginning of the LPA. The angles between these vectors were also recorded as parameters.

Statistical analysis of the parameters was also performed in MATLAB. The data was expressed as mean and standard deviation across patient groups and PA segments. The Kruskall-Wallis test was used to compare the various parameters across the multiple groups. Statistical significance was indicated by *p* values below 0.05.

### Virtual Deployment

With the measurements, stent sizes for the patient groups were proposed. Virtual deployment was performed by aligning the resized stent mesh file within an example patient PA to check for suitability.

## Results

Statistical analysis of the basic data of the study population (Table 1) showed statistically significant differences (*p* = 0.001) for the ages of the different patient cohorts. Height and weight of patients from the groups were not statistically significant (*p* > 0.1).

**Fig. 3:**
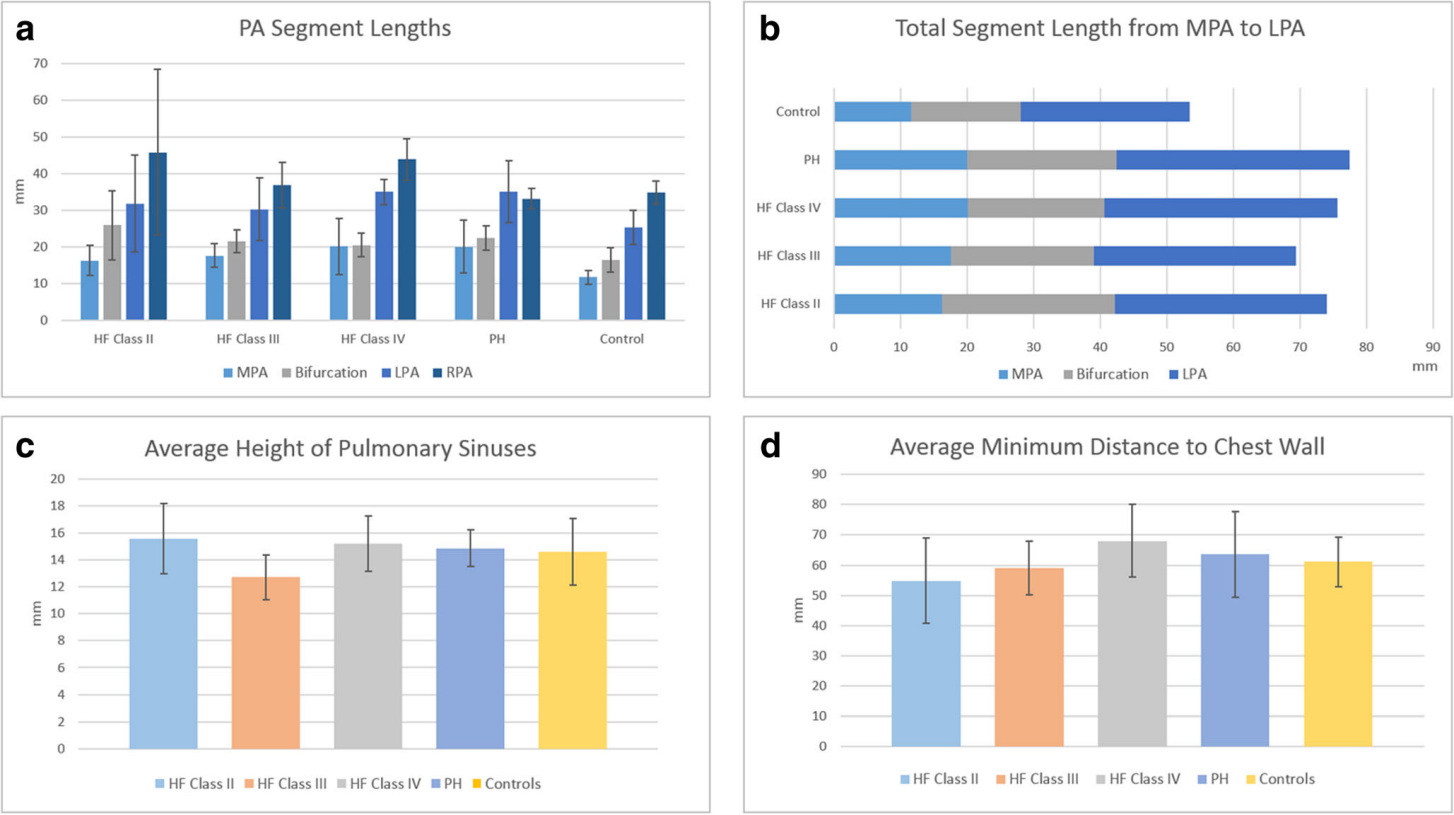
Measurements and analysis of the pulmonary artery segments for each group of patients based on the centreline. **a** Lengths of MPA, Bifurcation, LPA and RPA; **b** length from MPA to LPA; **c** average height of the pulmonary sinuses; **d** average minimum distance of closest MPA point to the chest wall

In Fig. 3, the average lengths of the PA centrelines of all subject groups are shown. In all subjects, the average length of the MPA was generally less than 20 mm, shorter than the current length of the SAWPS device stent. This suggests that the stent will need to be shorter than this or long enough to straddle the RPA bifurcation and be fixed in both the MPA and LPA. The average lengths of this bifurcation were 26.0 ± 9.5 mm for Class 2, 21.5 ± 3.1 mm for Class 3, 20.5 ± 3.2 mm for Class 4, 22.4 ± 3.0 mm for PH, and 16.5 ± 3.4 mm for Controls. The average lengths of the LPA were 31.8 ± 13.3 mm for Class 2, 30.3 ± 8.6 mm for Class 3, 35.1 ± 3.4 mm for Class 4, 33.3 ± 8.7 mm for PH, and 25.3 ± 4.7 mm for Controls. For completeness, the average RPA length was also calculated. These were 45.8 ± 22.6 mm for Class 2, 37.0 ± 6.2 mm for Class 3, 44.0 ± 5.7 mm for Class 4, 34.9 ± 4.8 mm for PH, and 34.9 ± 3.1 mm for Controls. When examined separately, only the lengths of the MPA were found to be statistically significant (*p* = 0.02). However, when examining the entire length of the MPA through to the LPA (Fig. 3b), this entire length was also statistically significant (*p* = 0.01).

Figure 3 also shows the average height of the pulmonary sinuses and the average minimum distances from the PA to the chest wall. The average heights were 15.6 ± 2.6 mm for Class 2, 12.7 ± 1.7 mm for Class 3, 15.2 ± 2.1 mm for Class 4, 14.9 ± 1.3 mm for PH, and 14.6 ± 2.5 mm for Controls. The average minimum distances from the PA to the chest wall were 54.9 ± 14.2 mm for Class 2, 59.1 ± 8.9 mm for Class 3, 68.1 ± 11.9 mm for Class 4, 63.6 ± 14.1 mm for PH, and 61.1 ± 8.2 mm for Controls. Both measurements were found to be statistically insignificant across patient groups (*p* > 0.05).

**Fig. 4:**
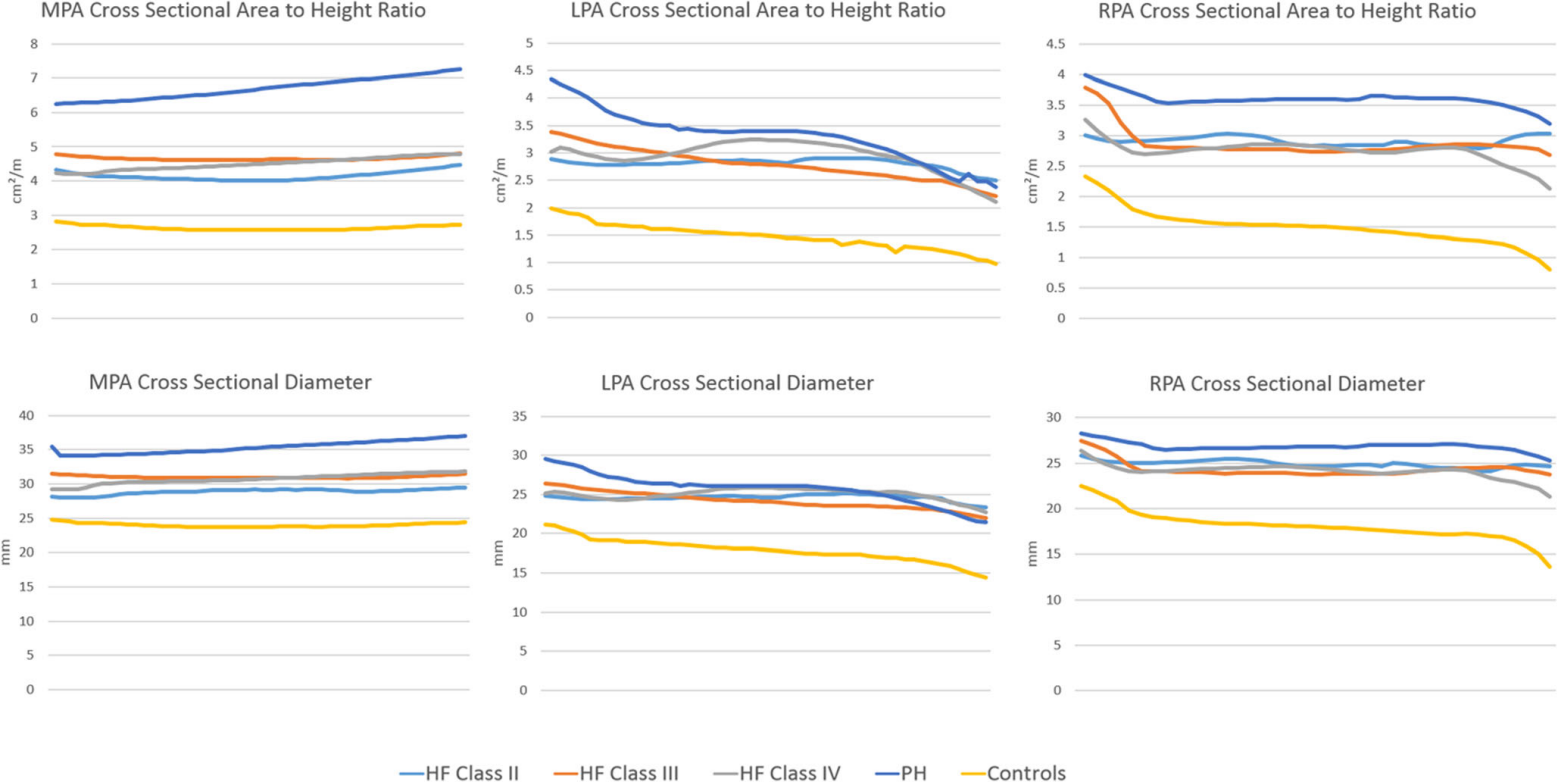
Average cross sectional area of PA segments along the centreline normalised to body height, in cm^2^/m (top row) and average values of the area-derived diameter (bottom row)—left to right: MPA, LPA and RPA. The x-axis in all graphs is the normalised length of the pulmonary artery sections

Figure 4 highlights the differences in the average cross sectional areas and diameters across the MPA, LPA and RPA of different patient cohorts. The top row shows the average cross sectional area of the PA lumen contour normalised to patient height (cm^2^/m) while the bottom row shows the average diameters of the same contours. The general trend over the average is greater cross sectional area and diameters for the PH group and lower parameters for the Controls. The average diameter from the MPA to the LPA, i.e. after the bifurcation, lowers by approximately 6 mm, indicating that the stent, if it is to straddle the bifurcation, needs to be flexible enough to adapt to the two diameters.

From Table 2, four landmarks can be seen marked on the PA on the left hand side: the start and end of the MPA, and the start and end of the LPA. When examining the cross sectional areas and diameters at these landmark points, statistical significance was found at the start and end of the MPA and the end of the LPA (*p* < 0.05). No statistical significance was found at the start of the LPA across the patient groups. These measurements will affect the diameter required of the stent.

**Fig. 5:**
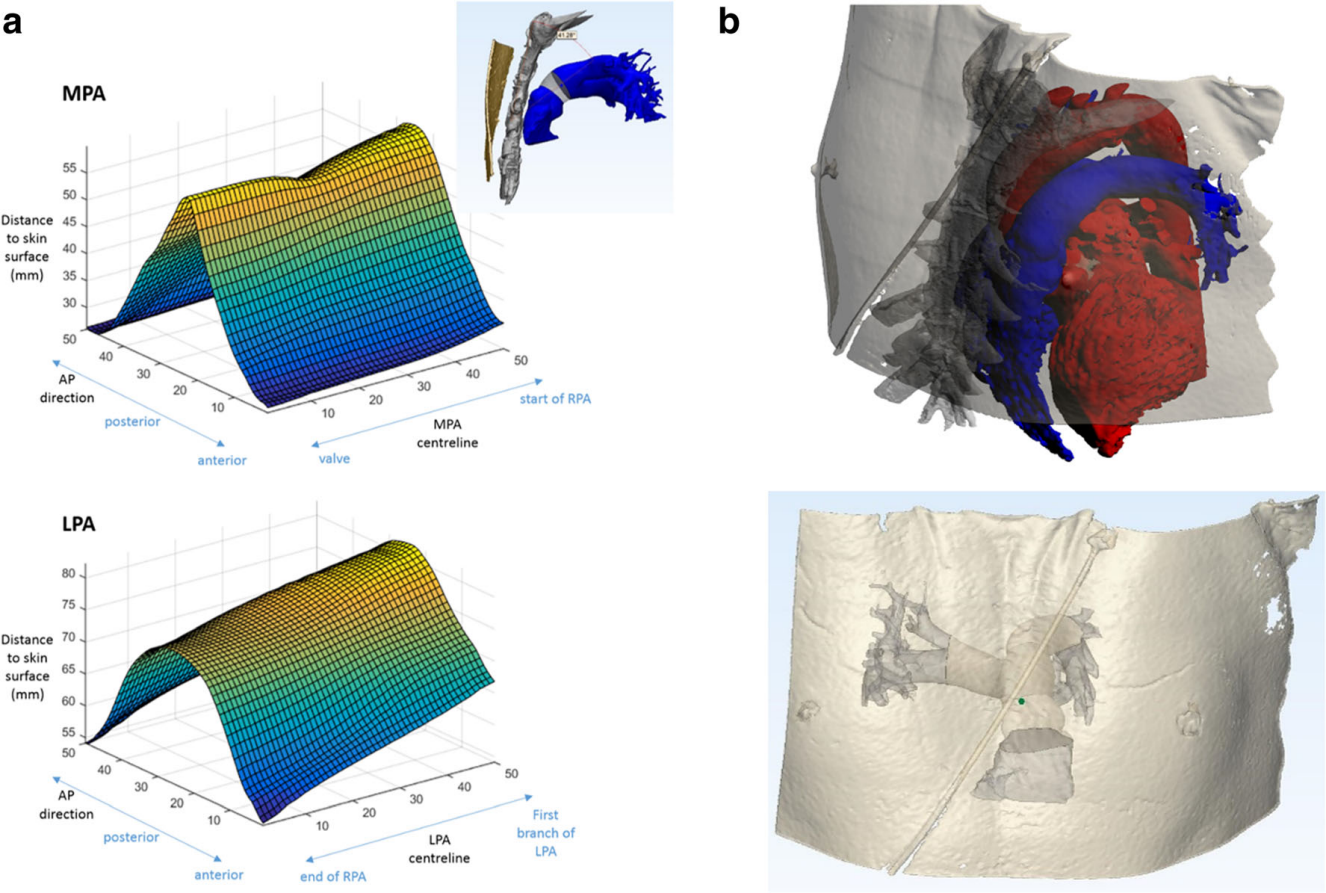
**a** Minimum distance to chest skin along MPA (from PV to RPA start) and LPA (from end of RPA to first LPA branch); **b** point on the centreline closest to the chest wall and corresponding chest wall point; the RPA and partially the LPA are hidden due to the aorta

Figure 5 shows one example of the distances of the skin surface from the MPA and LPA (a patient from NYHA Class 2). The graphs across all subjects resembled this, where the minimum distances to the chest wall were found along the anterior of the PA. As the RPA branches out at the posterior wall of the MPA-LPA, the anterior wall is the natural location for the placement of the device antenna. Distances to the skin surface were not found to be statistically significant between the patient groups (*p* > 0.05).

**Fig. 6:**
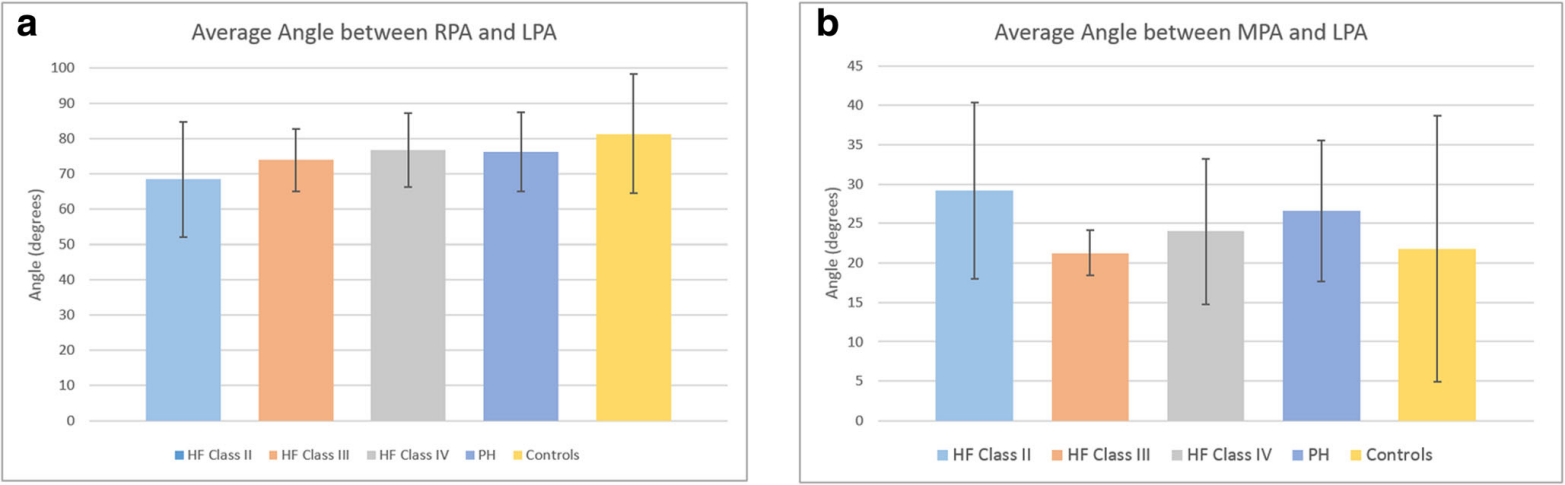
Angle between the segments after the bifurcation: **a** RPA and LPA, **b** MPA and LPA

Regarding the orientation of the PA segments with respect to each other, the angles between the RPA and LPA did not change much across the patient groups (Fig. 6). The average angle between the RPA and LPA were 68.4 ± 16.4° for Class 2, 74.0 ± 8.9° for Class 3, 76.8 ± 10.5° for Class 4, 76.3 ± 11.2° for PH, and 81.4 ± 16.8° for Controls. However, there were some differences between the MPA and LPA angle, with a large standard deviation observed between the Controls. The average angle between the MPA and LPA were 29.2 ± 11.2° for Class 2, 21.3 ± 2.9° for Class 3, 24.0 ± 9.3° for Class 4, 26.6 ± 8.9° for PH, and 21.8 ± 16.9° for Controls. Both angles were found to be statistically insignificant between patient groups.

**Fig. 7:**
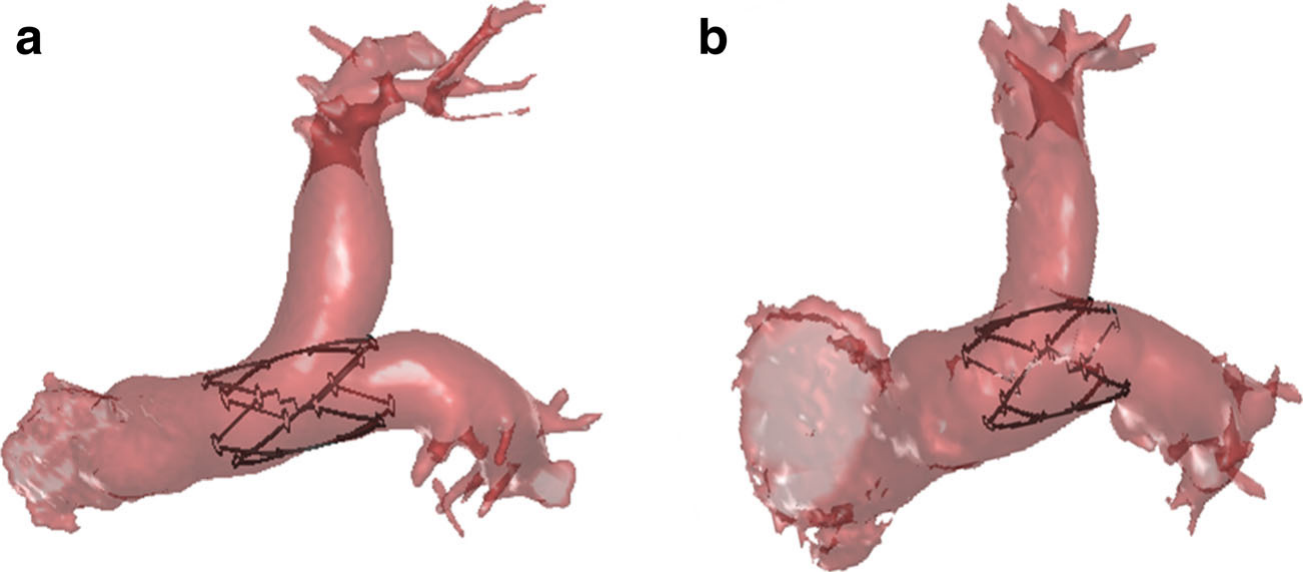
Virtual deployment of the proposed stent in the PA of **a** a NYHA Class 4 HF patient and **b** a PH patient

It is proposed that at least two separate stent sizes will be required to take into consideration the measurements found in this study. In particular, the length of the stent is recommended to be 45 mm for the NYHA Class 4 patients while 40 mm is sufficient for the HF patients. This is shown in Fig. 7.

## Discussion

The data presented in this paper was analysed to optimise the design of the SAWPS Device and could be used to select a range of different stent sizes to fit patient groups with similar PA morphology and spatial orientation. It will also guide clinicians to determine the most suitable position of the device within the PA and its branches.

The SAWPS device is a permanent implant and therefore consideration of the progression of the morphology of the PA is essential. The first design decision was that the whole device should rest against the PA wall and become endothelialised to reduce the risk of thromboembolism. An alternative approach has been taken by CardioMems [9] where a PA pressure sensor was placed in the LPA without regard to its position with respect to the vessel wall. They recorded no adverse events related to thromboembolism within the duration of the trial. The data on MPA area-derived diameter shows that a stent diameter chosen to fit a normal patient (c. 25 mm + oversizing) could be inadequate if the patient developed HF (c. 30 mm at III or IV) or PH (c. 35 mm). A self-expanding Nitinol design with a resting diameter of 40–45 mm with a radial strength sufficient to anchor the device during endothelialisation (6–12 weeks), but not too high to avoid the risks of stimulating exaggerated neointimal growth, vessel erosion and aneurysm formation [22, 23] would be ideal.

The longitudinal aspect and low profile of the device are well suited to lying against the inner wall of a tube. The antenna is flexible and will naturally rest against the wall if the vessel has a concave longitudinal surface, which is the case on the anterior surface of the MPA. The distal part of the antenna will be further held against the wall by the flowing blood; the proximal part needs to be held against the wall during endothelialisation to avoid generating vortices in the blood flow and the risk of emboli.

The stent can extend into the LPA or RPA—but given the significant discrepancy in size between the main PA and its branches it must be designed with different segmental radial sizes and strengths. The sharp angulation of the RPA means that the LPA is an easier choice for placement of the distal part of the device. The anterior wall from the MPA into the LPA is essentially a single concave surface. Extension across the bifurcation is permissible if the stent struts are spaced widely enough (open-cell design) to allow for unobstructed flow into the partially jailed right branch, as shown in Fig. 7. The length of the stent will need to be customised; the data suggests that a 40 mm stent length may not be sufficient for extension across the bifurcation in NYHA Class 4 HF patients and perhaps lengthening to 45 mm is necessary. Similarly, strut thickness needs to be minimised to reduce the risk of thrombus formation and/or flow alteration in the event of incomplete endothelialisation. Finally, the ideal stent would have a low profile that enables it to be delivered via a reasonably-sized highly-trackable sheath. Good imaging and good rotational registration between the catheter handle and tip will be necessary for optimal deployment.

Given the importance of placing the antenna as close as possible to the chest wall to enhance signal transmission, the antenna should be radiopaque to allow for optimal positioning and orientation within the pulmonary artery during implantation. For better wave propagation and RF signal noise reduction, positioning the device in the MPA segment is preferable. This is due to the fact that the MPA is the nearest segment to the sternum and chest wall and is not covered by other anatomical structures (the RPA segment is partially behind the ascending aorta). With optimal placement of the implant, there will be much greater tolerance on the optimal location of the ID antenna on the chest wall and more freedom to make an ergonomically-driven decision on placing the external antenna—for instance, within clothing.

At a recent initial preclinical test, an implantable SAWPS was delivered by an 18 F catheter into the main PA of a 75-kg swine via the femoral vein. The position and orientation of the device were adjusted using fluoroscopy and transoesophageal echocardiography before final deployment. The implanted sensor was interrogated and the signal quality was evaluated. The transmitting power and the received signal quality confirmed the previously measured performance of the implantable antenna with respect to the main PA orientation. The sensor orientation was observed to be parallel to the chest wall and the implantation depth was measured at 5 cm using the acquired images. The implanted sensor was interrogated for 30 min and the received signal was monitored in real time. The received signal quality was consistently good for about 25 min but started to deteriorate afterwards before the signal was completely lost at 30 min. The autopsy confirmed that migration of the implant into the LPA was the cause of signal loss. The migration occurred because the stent was undersized; the signal loss was due to the non-ideal orientation and depth of the LPA with respect to the chest wall in swine.

## Conclusion

Analysis has been performed on PA orientation and morphology from four patient groups and a control group and results show variance in PA segment lengths that will affect device placement. Initial preclinical tests confirmed the need for correct sizing and positioning of the device to ensure good received signal quality. These measurements will aid in stent design for future iterations of the SAWPS device and will ensure better management of patients with heart disease.

## Abbreviations

PA: Pulmonary artery
RPA: Right pulmonary artery
LPA: Left pulmonary artery
LV: Left ventricle
HF: Heart failure
HFpEF: Heart failure with a preserved ejection fraction
NYHA: New York Heart Association
ID: Interrogating device
BSA: Body surface area
SAWPS: Surface acoustic wave pressure sensor
FEM: Finite element modelling
CT: Computed tomography
MR: Magnetic resonance
PS: Pulmonary sinuses
